# The Changes of KCNQ5 Expression and Potassium Microenvironment in the Retina of Myopic Guinea Pigs

**DOI:** 10.3389/fphys.2021.790580

**Published:** 2021-12-23

**Authors:** Qin Yang, Qing Qing Tan, Chang Jun Lan, Bo Zhen Lv, Gui Mei Zhou, Wei Qi Zhong, Zhi Ming Gu, Yu Mei Mao, Xuan Liao

**Affiliations:** ^1^Department of Ophthalmology, Affiliated Hospital of North Sichuan Medical College, Nanchong, China; ^2^Department of Ophthalmology and Optometry, North Sichuan Medical College, Nanchong, China; ^3^The Translational Medicine Research Center and the Hepatobiliary Research Institute (North Sichuan Medical College), Nanchong, China; ^4^The Second Affiliated Hospital of Chongqing Medical University, Chongqing, China

**Keywords:** myopia, KCNQ5, potassium, M-current, Retigabine, XE991

## Abstract

KCNQ5 is suggestively associated with myopia, but its specific role in the myopic process has not been studied further. The aim of this study was to investigate the expression of potassium channel gene KCNQ5 and the changes of K^+^ microenvironment within the retina of form deprivation myopia (FDM) guinea pigs. A total of 60 guinea pigs were randomly divided into the normal control (NC) group, the self-control (SC) group, and the form-deprivation (FD) group for different treatments. Molecular assays and immunohistochemistry (IHC) were conducted to measure the expression and distribution of KCNQ5-related gene and protein in the retina. We determined the K^+^ concentration in the retina. In addition, the possible effects of form deprivation on potassium ionic currents and the pharmacological sensitivity of KCNQ5 activator Retigabine and inhibitor XE991 to the M-current in RPE cells were investigated using the patch-clamp technique. As a result, FD eyes exhibited more myopic refraction and longer AL. The mRNA and protein levels of KCNQ5 significantly decreased in the FD eyes, but the K^+^ concentration increased. In addition, the M-type K^+^ current [IK_(M)_] density decreased in FD RPE cells, and were activated or inhibited in a concentration-dependent manner due to the addition of Retigabine or XE991. Overall, KCNQ5 was significantly downregulated in the retina of FD guinea pigs, which may be associated with the increasing K^+^ concentration, decreasing IK_(M)_ density, and elongating ocular axis. It suggested that KCNQ5 may play a role in the process of myopia, and the intervention of potassium channels may contribute to the prevention and control of myopia.

## Introduction

Myopia is the most common refractive error in the world. The prevalence of myopia has shown a trend toward younger age and a significant increase, especially with the outbreak of COVID-19 in the past 2 years (Morgan et al., 2017; Wong et al., 2021). Previous studies have shown that refractive development is influenced by environmental, behavioral, and inherited factors (Flitcroft, 2012; Baird et al., 2020). Furthermore, the retina in myopic eyes undergoes changes at the morphological, anatomical, and molecular biological levels, suggesting that the occurrence and development of myopia may be closely associated with the retina (Flitcroft, 2012; Tkatchenko and Tkatchenko, 2019). Although some significant results have been obtained (Ang et al., 2014; Zhou et al., 2017; Ghyselinck and Duester, 2019; Zhao et al., 2020), the specific etiology and mechanism of the initiation and progression of myopia have not been clearly determined so far.

The association of KCNQ5 with refractive error and myopia has been identified by genome wide association study (Kiefer et al., 2013; Verhoeven et al., 2013; Li et al., 2021) and further verified by our previous study (Liao et al., 2017). KCNQ5, as a member of the KCNQ channel family, encodes the KCNQ5 channel subunit and participates in the transportation of potassium ions from the retina to the choroid (Verhoeven et al., 2013; Yoshikawa et al., 2014; Liao et al., 2017; Li et al., 2021). It has been reported that the potassium ion homeostasis in the subretinal space is critical to the physiological function of the eye, and disruption of this homeostasis may lead to the development of experimental myopia (Cases et al., 2017; Udensi and Tchounwou, 2017; Wu et al., 2020). Previous studies have shown that myopia may be related to dysregulation of potassium ion homeostasis in the retina and ciliary muscle (Crewther et al., 2006; Wu et al., 2020). Given that KCNQ5 encodes a potassium channel found in the RPE and neural retina, it is speculated that it may be involved in the ion transport mechanism underlying myopia.

KCNQ5, on the other hand, participates in the formation of voltage-gated M-channel (Biervert et al., 1998; Schroeder et al., 2000; Tzingounisa et al., 2010). The M-channel, composed of KCNQ2, KCNQ3, and KCNQ5, shows a characteristic time and voltage dependence, which has a key role in regulating the excitability of various central and peripheral neurons. However, the related molecules of the M-current in RPE remain unknown. Based on the regulation of KCNQ5 on the potassium microenvironment, we speculated that the change of KCNQ5 might affect the potassium ion homeostasis and M-current characteristics in the retina, thereby playing a role in myopia. To investigate this possibility, a series of *in vivo* and *in vitro* studies were conducted to characterize the changes of KCNQ5 channels and M-current manipulated by KCNQ5 in FDM guinea pig model.

## Materials and Methods

### Animals and Grouping

Three-week-old pigmented male guinea pigs (Cavia porcellus, 120–150 g) were supplied by Chongqing Tengxin Biotechnology Co., Ltd., Guinea pigs were raised at 25 ± 2°C with *ad libitum* access to food and water, and a 12-h light/12-h dark cycle (light from 8 a.m. to 8 p.m.) with approximately 300–500 lux (Li et al., 2014). The experimental group was monocularly fitted with translucent latex balloons for 4 weeks to induce form deprivation myopia (FDM), which included a self-control (SC) group and a form-deprivation (FD) group (*n* = 30 per group). The age-matched guinea pigs in the normal control (NC) group were left untreated (*n* = 30). In the form-deprivation group, the right eye was covered and the left eye, nose, mouth, and ears were exposed. The tightness and coverage of the facemask were checked every day (Lu et al., 2006). All animal experiments were approved by the Animal Care and Ethics Committee of North Sichuan Medical College (NSMC 202116), and followed the Statement of Association for Research in Vision and Ophthalmology (ARVO) for the Use of Animals in Ophthalmic and Visual Research.

In this study, refraction and axial length (AL) measurements were carried out prior to the start of treatment and 1, 2, 3, and 4 weeks after treatment. At the end of 4-week treatment, the guinea pigs were anesthetized with 3% sodium pentobarbital (50 mg/kg) until the cornea and pain reflex disappeared. The eyeballs were enucleated and positioned in a centrifuge tube (5 ml) and fixed in 4% paraformaldehyde for immunohistochemistry; Additional eyeballs were enucleated, then the cornea, lens, and vitreous were quickly removed on ice. The retinas were snapped frozen in liquid nitrogen, and stored at −80°C (Srinivasalu et al., 2018; Kole et al., 2020; Sghari et al., 2020).

### Measurement of Ocular Biometric Parameters

The ocular biometric parameters were measured using A-scan ultrasound (Cinescan, Quantel Medical, France) and retinoscopy (66 Vision-Tech Co., Ltd., China). Guinea pigs were given cycloplegic drops (compound tropicamide; Santen Pharmaceutical Co., Ltd., Japan) at 5-min intervals for three times to dilate pupils and inhibit ocular accommodation. Refractive error data was recorded by an independent experienced optometrist and analyzed using the spherical equivalent (spherical power + 1/2 cylindrical power) (McFadden et al., 2004; Yu et al., 2021).

The anterior chamber depth, lens thickness, and vitreous cavity length (the sum of the three parameters was AL) (Zhang et al., 2018) were measured with an A-ultrasound with an ultrasound probe frequency of 11 MHz, and the conduction velocities were set as 1557.5, 1723.3, and 1,540 m/s, respectively. Before measurement, topical anesthesia was performed with oxybucaine hydrochloride (Santen Pharmaceutical Co., Ltd., Japan); during measurement, the probe tip was perpendicular to the center of the cornea. Each measurement comprised an average of at least ten scans (Jiang et al., 2019; Yu et al., 2021).

### Quantification of KCNQ5 mRNA

Total RNA was respectively extracted from the retinas in three subgroups (*n* = 6 per subgroup, every two eyeball specimens in each group were pooled) using trizol reagent (Beyotime, Biotechnology, China) according to the manufacturer’s protocol. This was followed by reverse-transcription of 1 μg of total RNA to cDNA using Takara reverse transcriptase and the random primer provided in the kit (Takara, Japan). QPCR was performed on a Roche LightCycler instrument with Takara SYBR green II kit using resultant cDNA as template. The primer sequences were listed in Table 1. The protocol for the qPCR was an initial denaturation at 95°C for 30 s, followed by 40 cycles of denaturation at 95°C for 10 s, annealing at 59°C for 60 s, and extension at 97°C for 1 s. At the end of the amplification, calculating the relative expression of target genes in each group with the 2^–△△^ CT method (Ding et al., 2020; Wu et al., 2020).

**TABLE 1:** Primer sequences for quantitative PCR.

Primer name	Sequence (5′–3′)
KCNQ5	F: ACTTGGCTGGGAAGGTTGCTTTC
	R: GTGTTTCTGGCGGTGTTGTTCTTG
β-actin	F: CTGGGTATGGAATCCTGTGGCATC
	R: CAGCACTGTGTTGGCATAGAGGTC

### Determination of KCNQ5 Protein

The retinas from three subgroups (*n* = 6 per subgroup, every two eyeball specimens in each group were pooled) were covered with RIPA lysis buffer containing 1 mM of PMSF, and the protein was extracted on ice. This was followed by the centrifugation at 12,000 rpm/min for 10 min to collect the supernatants for western blot analysis. BCA method was used to detect the protein concentration. The samples mixed with 5 × loading buffer were then placed in 70°C for 15 min, electrophoresed under reducing conditions on 8% SDS-PAGE gels, and electrotransferred onto a nitrocellulose membrane. After blocking with 5% non-fat milk at room temperature for 2 h, the primary antibody of KCNQ5 (1:500; Invitrogen, United States) and β-tubulin (1:5,000; Beyotime Biotechnology, China) were incubated with the membranes at 4°C overnight. After washing 3 times for 10 min each with TBST, the membranes were incubated with goat anti-rabbit secondary antibody (1:10,000; Boster, China) at room temperature for 1 h, and then washed again with TBST for 3 times. Finally, protein bands were visualized using the FUSION-FX7 imaging system (Vilber Lourmat, France) and the optical density of the bands was analyzed using Image J software. All western blots shown are representative of at least three independent experiments (Harper et al., 2016).

### Immunohistochemistry of KCNQ5

The eyeballs of three subgroups (*n* = 6 per subgroup, every two eyeball specimens in each group were pooled) were, respectively, fixed with 4% paraformaldehyde, then embedded in paraffin and cut into 4 μm sections. The histological sections were incubated with 3% hydrogen peroxide-methanol solution at 37°C for 15 min, and then rinsed with PBS 3 times. Paraffin sections were blocked with 3% goat serum for 30 min, and then incubated with primary antibody (KCNQ5; 1:500, Millipore, United States) or negative control (PBS) overnight at 4°C. On the second day, the sections were taken out following reheating for 30 min, washed with PBS 3 times, and then dropped with secondary antibody (Boster, China) and incubated at 37°C for 1 h. The DAB reagent (ZSGB-BIO, China) was subsequently used to detect the immunoactivity. Then the sections were counterstained with hematoxylin (Boster, China), and dehydrated in different concentrations of ethanol. Finally, after the sections were sealed with neutral resin, images were captured using an optical microscope (Caminos et al., 2015; Dong et al., 2020).

### Detection of Potassium Ion Concentration

The retinas of three subgroups (*n* = 6 per subgroup) were collected and homogenized with sterile saline (*w/v* = 1:9), followed by centrifugation (2,500 rmp/min, 4°C, 10 min) for total K^+^ concentration determination. Then K^+^ of supernatant was measured using a K^+^ detection kit (Nanjing Jiancheng Bioengineering Institute, China) according to the manufacturer’s instructions. Meanwhile, the protein concentration of the supernatant was measured by the BCA method. Absorbance values were determined at 450 nm using a microplate reader (Unico (Molecular Devices SpectraMax250, United States) (Tian et al., 2019; Liao et al., 2021).

### Retinal Pigment Epithelium Primary Cell Culture and Patch Clamp Record

#### Cell Isolation and Culture

After the guinea pigs were sacrificed, the eyeballs were enucleated immediately, washed with physiological saline, and stored at 4°C. Then they were soaked in iodophor for 5 min × 2 times, 75% alcohol for 5 min × 2 times, and normal saline for 5 min × 2 times. Anterior segment tissues and retinas were gently removed; the eye cups were washed in chilled PBS, and then digested with 0.25% trypsin digestion solution (Gibco, United States) at 37°C for 30 min. Then digestion was terminated with fresh DMEM/F12 (Gibco, United States) medium containing 20% fetal bovine serum. The cell pellet was resuspended in the DMEM/F12 medium and centrifuged at 1,000 rpm/min for 10 min. After the supernatant was discarded, the suspended RPE cells were inoculated on the culture dish. The culture medium was changed every 2–3 days and cells were passaged as they reached confluence (Zhang et al., 2011; Yang et al., 2019; Xin et al., 2021). The fourth-generation primary cells were used in the follow-up experiments, and the cytoplasmic pigment particles of the cells were reduced; the cells tended to be transparent at this stage.

#### Drug and Solution Preparations

The standard bath solution (HEPES-Ringer) consisted of (in mM) 144 NaCI, 2.5 KCl, 2 CaCl_2_, 0.5 MgCl_2_, 5HEPES, and pH was adjusted to 7.2–7.4. The standard pipette solution was (in mM) 30 KCl, 83 K-gluconate, 10 K_2_HEPES, 5.5K_2_EGTA, 0.5 CaCl_2_, and 4 MgCl_2_, 10 glucose, and pH was adjusted to 7.2–7.4. Each solution was filtered with a disposable syringe filter (0.22 μm, Millipore, United States) on the day of use. Retigabine (KCNQ5 activator, selleckchem, United States) and XE991 (A KCNQ5 blocker, Sigma, United States) were dissolved in DMSO to prepare mother liquor. All drugs and reagents were aliquoted and stored at −20°C, and diluted 1,000 times with normal saline when used (Pattnaik and Hughes, 2012).

#### Patch-Clamp Electrophysiology

Different types of ionic currents were measured in the whole-cell mode using the standard patch-clamp technique with an Axopatch 200B patch-clamp amplifier (Molecular Devices, Sunnyvale, CA, United States), which was interfaced to a PC running pCLAMP suite software. The liquid junction potentials were corrected shortly before seal formation was established. The resistances between the standard pipette and the bath solution were about 6–8 MΩ. The whole cell current recording was performed at room temperature, and the RPE cells were sealed by adjusting the three-dimensional manipulator. When the sealing impedance reached gigaohm seals by slightly touching the basal membrane of a cell with the pipette tip, the membrane patch was then ruptured by a slightly stronger suction pulse to form a whole-cell recording mode. After the capacitance and series resistance were compensated, the membrane potential was clamped at −20 mV, and a 1-s depolarizing pulse of −60 mV was delivered to activate the M-current. The M-current density was expressed as the ratio of the current amplitude to the membrane capacitance. Data acquisition and analysis were carried out using pCLAMP 10.0 (Wen et al., 1993; Hughes and Takahira, 1996; Pattnaik and Hughes, 2012; Yang et al., 2019).

### Statistical Analysis

All data was expressed as mean ± standard deviation (SD), and statistical analysis was performed using the statistical software SPSS 25.0; GraphPad Prism 8.0 and Oringin 8.5 were used for graphing and data curve fitting. A paired-sample *t-*test was used for comparisons in biological parameters between the FD and SC eyes of the experimental group. Two-way repeated measures analysis of variance was used to compare the biological parameters between FD group and NC group at different time points. One-way analysis of variance was used to compare the differences in the molecular level of each group. LSD-*t*-test was used for multiple comparisons between groups. *P* < 0.05 was considered statistically significant.

## Results

### Changes in Refraction and Axial Length

**FIGURE 1:**
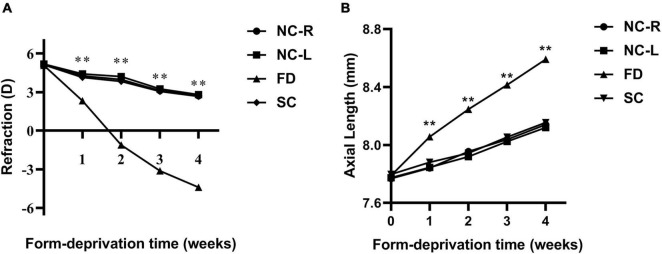
Changes in refraction **(A)** and AL **(B)** in eyes of form deprivation guinea pigs. (**P < 0.01. FD, the right eye of the experimental group; SC, the left eye of the experimental group; NC-R, normal control right eye; NC-L, normal control left eye).

Prior to myopic induction, the refractive states of the eyes of the 3-week-old guinea pigs were mostly hyperopia, and there was no significant difference in refraction and AL among groups (all *P* > 0.05). From the first week of treatment, the refraction of FD eyes in the FD group gradually increased and was significant higher than those in the SC group and the NC group (all *P* < 0.01); Meanwhile, it was noted that the AL of the form-induced eyes in FD group significantly increased compared with those in the SC group and the NC group after treatment (all *P* < 0.01). There was a significant myopic shift in refractive error in the FD group after 2 weeks of treatment. After 4 weeks of treatment, the refraction of eyes in the FD group increased to −4.41 ± 1.16D, and that in the SC and NC group were 2.72 ± 1.19D and 2.67 ± 1.07 D, respectively (all *P* < 0.01). Correspondingly, the AL was 8.59 ± 0.10 mm vs. 8.14 ± 0.11 mm vs. 8.15 ± 0.12 mm in the three groups (all *P* < 0.01; Figure 1).

### Relative Expression of KCNQ5 mRNA

**FIGURE 2:**
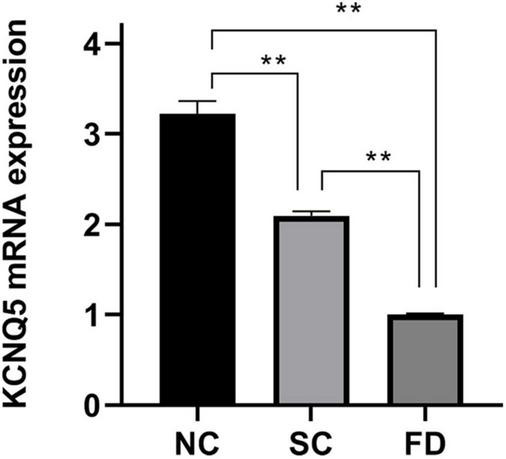
Measurement of KCNQ5 expression at mRNA level after 4 weeks of treatment. (Histogram analysis indicated that the KCNQ5 mRNA level in FD group significantly decreased after 4 weeks of form deprivation. **P < 0.01).

The results showed that KCNQ5 mRNA level in retinas in FD guinea pigs was significantly lower than that of SC and NC guinea pigs after 4-week myopic induction (*P* < 0.01, Figure 2). Representative results of at least three independent experiments are shown.

### Expression of KCNQ5 Protein

**FIGURE 3:**
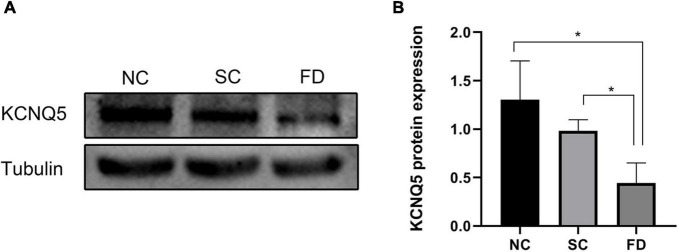
Measurement of KCNQ5 expression at protein levels after 4 weeks of treatment. **(A)** Showed the western blot electrophoretic bands of each group; **(B)** showed the statistical results of gray level of the stripe (*P < 0.05).

Western blot experiments showed that the expression of KCNQ5 in the covered eyes of the FD group after 4 weeks of form deprivation was significantly lower than those of the SC group and the NC group (*P* = 0.021); There was no statistically significant difference between the two control groups (*P* = 0.196; Figures 3A,B). This indicated that the expression of KCNQ5 protein was decreased in FDM.

### Distribution and Expression of KCNQ5

**FIGURE 4:**
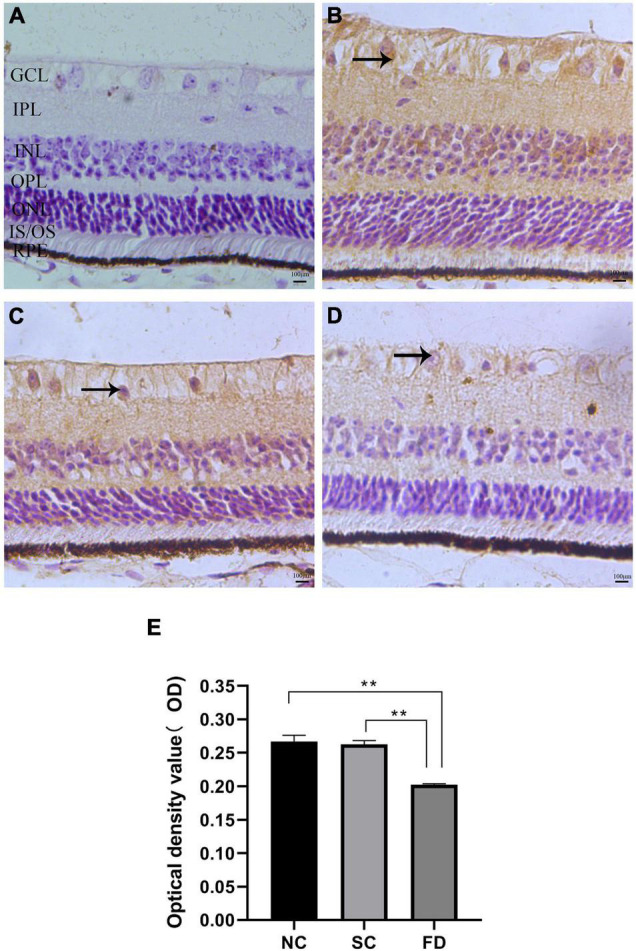
Expression and distribution of KCNQ5 in each layer of the retina. **(A–D)** Showed the immunohistochemical staining results of each group; **(E)** showed the statistical results of the average optical density of the immunohistochemical images. **(A)** Negative control, the staining after incubation with PBS; **(B)** the NC group; **(C)** the SC group; **(D)** the FD group; GCL, ganglion cell layer; IPL, inner plexiform layer; INL, inner nuclear layer; OPL, outer plexiform layer; ONL, outer nuclear layer; IS/OS, inner and outer segments of photoreceptor; RPE, pigment epithelial layer. The positive expression of KCNQ5 in the retina was brown. The scale was 100 μm and the magnification was 400 times (**P < 0.01).

The results of immunohistochemistry showed that KCNQ5 protein molecules were positive in the RPE layer, the inner and outer segments of the photoreceptor, the outer plexiform layer, the inner plexiform layer, and the ganglion cell layer of the guinea pig retina (Figure 4A). Compared with the control group at the corresponding time point, the expression of KCNQ5 in the FD group showed a downward-regulated trend, and the differences were statistically significant (*P* < 0.01; Figure 4B).

### Changes in Potassium Ion Concentration

**FIGURE 5:**
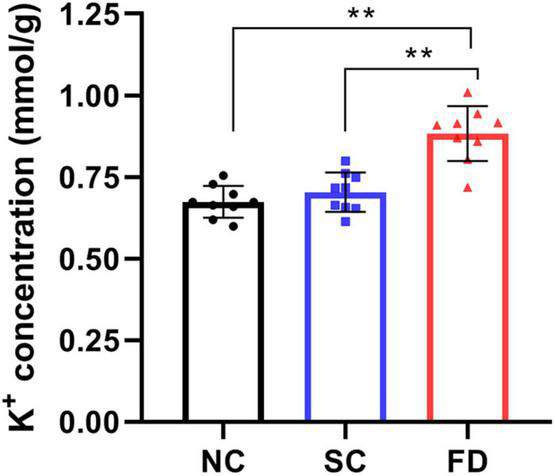
Measurement of K^+^ concentration in retinas after 4 weeks of treatment. The result indicated that the K^+^ concentration in FD guinea pigs were higher than those in NC and SC guinea pigs (**P < 0.01).

To investigate the effect of FDM on K^+^ concentration, we measured the K^+^ concentration of retinas using the commercial kit. The result showed that the K^+^ concentration significantly increased in retinas of FD guinea pigs compared with those control guinea pigs after 4 weeks of treatment (*P* < 0.01). There was no statistically significant difference between the NC group and the SC group (*P* = 0.622; Figure 5).

### Effect of Form Deprivation Myopia on M-Type K^+^ Current

**FIGURE 6:**
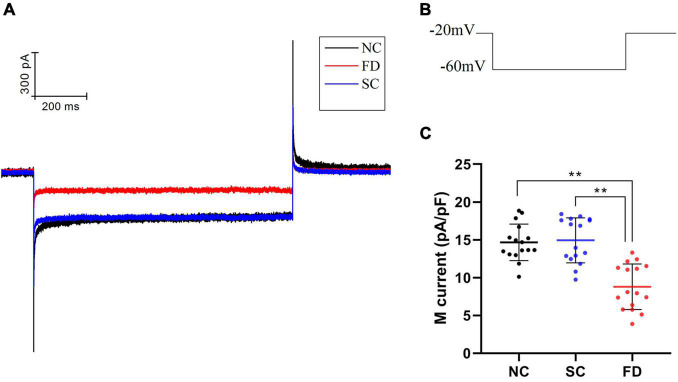
M-currents of RPE cells in each group. **(A)** Original diagram of M-current of RPE cells in each group. **(B)** Protocol for recording M-current. **(C)** Statistical graph of the I_K(M)_ density of each group (**P < 0.01).

**FIGURE 7:**
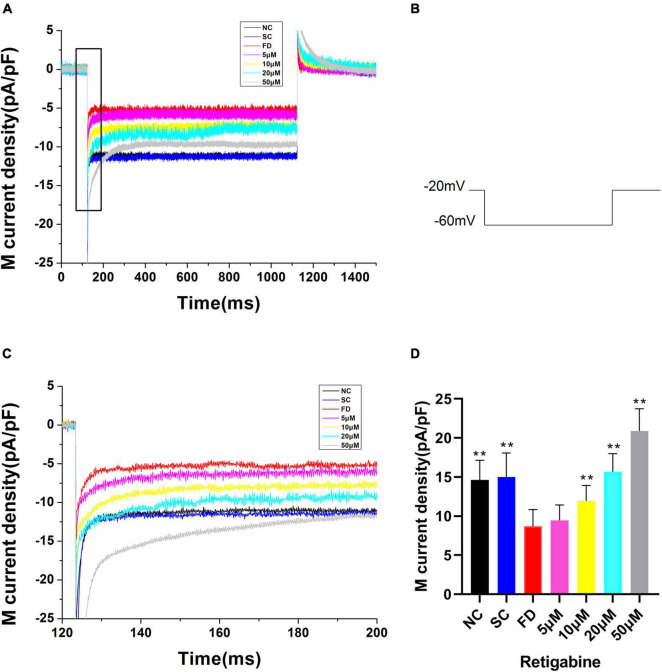
M-currents treated with Retigabine in the FD group. **(A)** I_K(M)_ Density of RPE cells treated with different concentrations of Retigabine. (a) Black, before administration; (b) Red, 5 μM; (c) Blue, 10 μM; (d) Purple, 20 μM; (e) Gray, 50 μM. **(B)** Protocol for recording M-current. **(C)** The expanded recordings from the dashed box in **(A)**, and indicated the effect of Retigabine on the I_K(M)_ density. **(D)** Statistical graph of the I_K(M)_ density of each group. When the concentration increased to 10, 20, and 50 μM, the differences were statistically significant (**P < 0.01).

**FIGURE 8:**
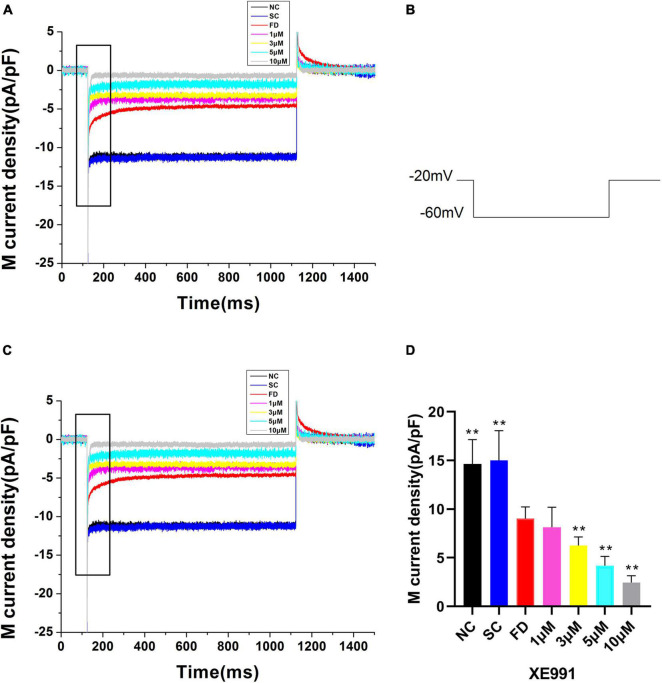
M-currents treated with XE991 in the FD group. **(A)** I_K(M)_ density of RPE cells treated with different concentrations of XE991. (a) Black, before administration; (b) Red, 1 μM; (c) Blue, 3 μM; (d) Purple, 5 μM; (e) Gray, 10 μM. **(B)** Protocol for recording M-current. **(C)** The expanded recordings from the dashed box in **(A)**, indicated the effect of XE991 on the I_K(M)_ density. **(D)** Statistical graph of the M-current density of each group. When the concentration increased over 3μM, the differences were statistically significant (**P < 0.01).

Compared with the control group, the I_K(M)_ density in RPE cells of the FD group was decreased. And the difference was statistically significant (*P* < 0.01). There was no statistically significant difference between the two control groups (*P* = 0.785; Figure 6). *In vitro* experiments (Figure 7) showed that the Retigabine increased the I_K(M)_ density of the FD group and resulted in a differential activation of the I_K(M)_ in a concentration-dependent manner. The difference was not statistically significant while the concentration of Retigabine was below 10 μM, while under the concentration of 20 μM, the M-current density increased and even restored to a level similar to that of the control group. As depicted in Figure 8, the XE991 reduced the I_K(M)_ density in a concentration-dependent manner. And the KCNQ5 current was relatively sensitive to block by XE991. At the XE991 concentrations over 3 μM, the inhibition was obvious.

## Discussion

Potassium plays a vital role in the maintenance of normal cell functions such that any imbalance in potassium ion may adversely affect health. KCNQ5 is a key regulator of potassium ion transport which is important to the ion transportation between RPE and choroid. Its activity can be affected by specific channel modulators (Zhang and Wildsoet, 2015). The present study provided evidence of the KCNQ5 expression in the retina of guinea pig and demonstrated that the M-current of RPE cells was mediated by KCNQ5 channel. Furthermore, both retinal KCNQ5 expression and I_K(M)_ decreased after induction of myopia with form deprivation, indicating the KCNQ5 involvement in myopia development in the guinea pig.

Studies in monkey, rat, and bovine have shown that both RPE and neuroretina express KCNQ5 (Zhang et al., 2011; Pattnaik and Hughes, 2012; Zhang and Hughes, 2013; Caminos et al., 2015); Our study further showed that KCNQ5 was mainly distributed in RPE layer, inner and outer segment of photoreceptor, outer plexus layer, inner plexus layer, and ganglion cell layer in the retina of guinea pig by immunohistochemistry detection. We found that the distribution of KCNQ5 in the retina is similar to that reported by previous studies (Zhang et al., 2011; Caminos et al., 2015). Additionally, our study showed that both transcription and protein levels of KCNQ5 were lower in the FD group than those in the NC and the SC groups (Figures 2–4), which may indicate that the function of the KCNQ5 was inhibited to a certain extent in the FDM. In the results of qPCR, compared with the NC group, the mRNA of KCNQ5 was decreased (Figure 2) but the protein was similar (Figures 3, 4) in the SC group. This result can be interpreted as follows: the relationship between mRNA and protein is not strictly linear, but as a result of complex processes (Abreu et al., 2009; Buccitelli and Selbach, 2020). Therefore, the down-regulation of the expression at the mRNA level does not mean down-regulated expression at the protein level. We thought that the low expression of KCNQ5 transcription level in SC group may be related to the stress reaction caused by form deprivation or the poor stability of RNA, but the expression of protein levels was not affected. The above experimental results demonstrated the differential expression of KCNQ5 in myopia for the first time, and further explained the possibility of the KCNQ5’s participation in the regulation of myopia in guinea pigs.

In order to explore the specific pathways of KCNQ5 involved in myopia, we focused on the role of KCNQ5 in potassium ion transport, and speculated that it might affect the development of myopia by regulating the potassium ion transport state in the retina. Recently, Wu et al. (2020) found the decreased expression and activity of Na^+^/K^+^-ATPase in ciliary muscles of lens-induced myopia guinea pigs, which leads to the disturbance of potassium ion homeostasis. This not only provided evidence for our speculation, but also further suggested that changes in the potassium microenvironment may be related to the pathogenesis of myopia. In the present study, we measured the K^+^ concentration in the retina and found that it was up-regulated in FDM. Crewther et al. (2006) have raised the issue of potassium ion changes in myopia and the results showed that potassium ion concentration in the photoreceptor outer segment, RPE, and the outer part of the retina of FDM chicks increased, which was consistent with ours.

Participating in the formation of M-current was another feature of KCNQ5 as an M-channel, characterized by a low threshold voltage (about −60 mV), and exhibited slow activation and inactivation kinetics (Browna and Yu, 2000; Zhang et al., 2011). M-current shows a continuous outward rectification characteristic which is regulated by specific channel inhibitors and activators (Delmas and Brown, 2005; Yang et al., 2019). In the electrophysiological study of patch clamp in guinea pig RPE cells, we found that M-current may be regulated by KCNQ5, and suppressed in FDM. KCNQ5 can be activated and inhibited by the specific KCNQ5 channel activator Retigabine and inhibitor XE991, which were selected to continue the *in vitro* cell experiment. Our results showed that the M-current of the FD group restored to a level similar to that of the control group under the action of Retigabine, while it was further inhibited by XE991. It can be explained that, at the physiological level, the function of the M-channel was inhibited in FDM, which may lead to a decrease in the IK_(M)_. As a consequence, the conduction process of electrochemical signals in the retina was affected, thereby the processing and transmission of visual signals in the retina were blocked. Ultimately, refractive development of the eye was influenced. The above approach can also be one of the ways that KCNQ5 participates in the regulation of refractive development.

The increase of K^+^ concentration and decrease of IK_(M)_ in retinas may at least partly be explained by the decrease of KCNQ5, resulting in an obstruction of K^+^ transportation from the retina to the choroid. This change in the microenvironment might affect the ocular ion homeostasis and normal development, but the specific mechanism still needs further research. The above research results suggest that KCNQ5 may participate in the development of myopia by regulating changes in the potassium ion level including the retinal potassium ion concentration and IK_(M)_.

Several limitations of this study should be considered. The number of animals was relatively small from a statistical point of view, the K^+^ concentration in the choroid was not collected during the experiment; and the design scheme for the electrophysiology needs to be improved. In addition, M current may be regulated by other KCNQ subunits (such as KCNQ2 and KCNQ3). This study did not detect the expression of these KCNQ subunits. Further *in vivo* drug intervention experiments may help improve the mechanism of KCNQ5 involvement in myopia. In the future research, such limitations should be addressed.

## Conclusion

In summary, we investigated the expression and distribution of KCNQ5 in the retina, and the results demonstrated a down-regulation in FDM. In addition, FDM also caused a significant increase in K^+^ concentration of retina. The I_K(M)_ density in the FD group was lower than those in control groups, which could be activated or inhibited by Retigabine or XE991. Collectively, our study indicated that KCNQ5 may participate in form-deprivation myopia, but the specific mechanisms remain to be further studied.

## Data Availability Statement

The datasets presented in this study can be found in online repositories. The names of the repository/repositories and accession number(s) can be found at: https://www.ncbi.nlm.nih.gov/, XM_003478277.3.

## Ethics Statement

The animal study was reviewed and approved by the Animal Care and Ethics Committee of North Sichuan Medical College.

## Author Contributions

XL designed, supervised the study, and acquired funding. QY performed the animal and *in vitro* experiments and analyzed the data, and wrote the original draft. XL, QT, and CL reviewed and revised the manuscript. BL, GZ, WZ, ZG, and YM managed the project samples and data. All authors approved the final version of the manuscript.

## Conflict of Interest

The authors declare that the research was conducted in the absence of any commercial or financial relationships that could be construed as a potential conflict of interest.

## Publisher’s Note

All claims expressed in this article are solely those of the authors and do not necessarily represent those of their affiliated organizations, or those of the publisher, the editors and the reviewers. Any product that may be evaluated in this article, or claim that may be made by its manufacturer, is not guaranteed or endorsed by the publisher.

## Abbreviations

FDM: form deprivation myopia
NC: normal control
SC: self-control
FD: form-deprivation
QPCR: Quantitative polymerase chain reaction
PBS: phosphate-buffered saline
IHC: immunohistochemistry
AL: axial length
GCL: ganglion cell layer
IPL: inner plexiform layer
INL: inner nuclear layer
OPL: outer plexiform layer
ONL: outer nuclear layer
IS/OS: inner and outer segments of photoreceptor
RPE: retinal pigment epithelium
I_K(M),_: M-type K^+^ current
SD: standard deviation.
